# Masking discolored substrates with resin composites: effect of layering strategies

**DOI:** 10.1590/0103-6440202405910

**Published:** 2024-09-16

**Authors:** Pablo Machado Soares, Gratcheva Falcão Chiapinotto, Atais Bacchi DDS, Gabriel Kalil Rocha Pereira

**Affiliations:** 1 Post-Graduate Program in Oral Sciences (Prosthodontics Units), Faculty of Dentistry, Federal University of Santa Maria(UFSM), Santa Maria, RS, Brazil.; 2 Faculty of Dentistry, Federal University of Santa Maria(UFSM), Santa Maria, RS, Brazil.; 3 Professor, MSciD Post-Graduate Program in Dentistry, Paulo Picanço School of Dentistry(FACPP), Fortaleza, CE, Brazil.

**Keywords:** Composite Resin, color, dental materials, dental aesthetics

## Abstract

This study aimed to evaluate the masking ability of different resin composite (RC) layering techniques over discolored substrates. Layering strategies were tested (n=10), using different RCs: flowable opaque, white dentin, A1 dentin, A1 body, and A1 enamel (Filtek Z350XT; 3M ESPE). Bilayer and trilayer RC combinations resulted in final thicknesses of 1 mm, 1.5 mm, and 2 mm. Substrates tested were: A1 (reference), A3, A4, B3, C2, and C4 (Filtek Z350XT Dentin; 3M ESPE). Color differences (∆E00) were measured for the RC layers over discolored substrates with the CIEDE2000 formula. The results were compared statistically (One-way ANOVA) and descriptively (acceptability=1.77 and perceptibility=0.81 thresholds). The layering strategy influenced the ∆E00 of RCs over all substrates (P<0.001). The 1 mm bilayer group combining 0.5 mm of dentin and 0.5 mm of enamel led to ∆E00 below AT for substrates A3 and B3; the 1.5 mm bilayer group combining A1 dentin (1 mm) and enamel (0.5 mm) provided ∆E00 below AT for substrates A3, A4, and C2 and ∆E00 below PT for B3; for substrate C4, the 2 mm trilayer group combining flowable opaque (0.2 mm), A1 dentin (1.3 mm) and enamel (0.5 mm) provided ∆E00 below PT, and the 1.5 mm trilayer groups (flowable opaque + 0.8 mm dentin or body + enamel) led to ∆E00 below AT. Resin Composites were effective in masking discolored substrates. The most adequate layering strategy depended on substrate shade.

## Resumo

O objetivo deste estudo foi avaliar a capacidade de mascaramento de diferentes técnicas de estratificação de resina composta (RC) sobre substratos descoloridos. Estratégias de estratificação foram testadas (n=10), utilizando diferentes RCs: opaco fluido, dentina branca, dentina A1, corpo A1 e esmalte A1 (Filtek Z350XT; 3M ESPE). As combinações RC bicamada e tricamada resultaram em espessuras finais de 1 mm, 1,5 mm e 2 mm. Os substratos testados foram: A1 (referência), A3, A4, B3, C2 e C4 (Filtek Z350XT Dentina; 3M ESPE). As diferenças de cor (∆E_00_) foram medidas para as camadas RC sobre substratos descoloridos com a fórmula CIEDE2000. Os resultados foram comparados estatisticamente (ANOVA de 1 fator) e descritivamente (aceitabilidade=1,77 e perceptibilidade=0,81 limiares). A estratégia de estratificação influenciou o ∆E_00_ dos RCs em todos os substratos (P<0,001). O grupo de bicamada de 1 mm combinando 0,5 mm de dentina e 0,5 mm de esmalte levou a um ∆E_00_ abaixo de AT para os substratos A3 e B3; o grupo de bicamada de 1,5 mm combinando dentina A1 (1 mm) e esmalte (0,5 mm) forneceu um ∆E_00_ abaixo de AT para substratos A3, A4 e C2 e ∆E_00_ abaixo de PT para B3; para o substrato C4, o grupo tricamada de 2 mm combinando opaco fluido (0,2 mm), dentina A1 (1,3 mm) e esmalte (0,5 mm) forneceu um ∆E_00_ abaixo de PT, e os grupos tricamada de 1,5 mm (opaco fluido + dentina 0,8 mm ou corpo + esmalte) levou a um ∆E_00_ abaixo de AT. As resinas compostas foram eficazes para mascarar substratos descoloridos. A estratégia de estratificação mais adequada dependeu da tonalidade do substrato.

## Introduction

The esthetical outcome is one of the major concerns in restorative dentistry. Nowadays, the natural appearance of the teeth influences the patient's well-being and quality of life ^1^. The presence of discolored substrates is common in clinical practice, usually due to trauma, endodontic complications, and enamel/dentin developmental alterations ^2^. Therefore, restorations over discolored tooth substrates are often necessary, which challenges the esthetical predictability of the final treatment ^3^.

The resin composite (RC) layering technique is widely used in clinical practice considering its proper cost-benefit, adequate esthetical results, and satisfactory restoration longevity ^4^ ^,^ ^5^. In addition, RC restorations might allow conservative tooth preparations, depending on the clinical scenario ^4^ ^,^ ^5^. The RC layers must mimic the optical properties of tooth structures and provide a natural appearance ^6^ ^,^ ^7^ ^,^ ^8^. In this sense, factors such as RC chroma, hue, translucency and lightness, restoration thickness, and substrate shade must be considered ^2^ ^,^ ^9^ ^,^ ^10^ ^,^ ^11^ ^,^ ^12^.

A wide range of material translucencies is available in the market, such as flowable opaques, dentin, the ‘body’ RC (considered a universal restorative, being more translucent than dentin and less translucent than enamel), and enamel ^13^ ^,^ ^14^ ^,^ ^15^ ^,^ ^16^. These characteristics also influence the lightness of the material. Hence, different combinations of these RC translucencies may influence the color differences of restorations ^14^ ^,^ ^17^. For example, studies showed that the application of opaque RCs as first layers improves the masking ability of restorations over discolored substrates ^3^ ^,^ ^6^ ^,^ ^11^ ^,^ ^18^ ^,^ ^19^.

The thickness of the restorative material has also a significant impact on light transmission ^9^ ^,^ ^11^ ^,^ ^20^ and, consequently, it influences the color differences over discolored substrates ^21^. A previous scoping review ^21^ ^)^ indicated that opaque RC restorations of 1 to 2 mm of thickness or layering techniques are necessary to mask discolored substrates. Even so, there is still no consensus about the definition of predictable RC restoration protocols for different levels of tooth discoloration when considering the layering strategy.

Thus, considering the aforementioned factors, this study aimed to evaluate the effect of different RC layering techniques on the masking ability of discolored substrates. This study hypothesized that the RC layering strategy would significantly influence the color differences over discolored substrates.

## Materials and methods

The experimental design of the present study is depicted in Table 1, as the description of the evaluated groups.

**Table 1:** Experimental design.

Groups	Resin composite layers and thickness	Substrates shade	Outcome
D0.5+B0.5	A1 dentin and A1 body (0.5 mm each); total: 1.0 mm	A1 (Reference); A3; A4; B3; C2; C4	Color difference (∆E_00_)
D0.5+E0.5	A1 dentin and A1 enamel (0.5 mm each); total: 1.0 mm		
D0.5+B0.5+E0.5	A1 dentin, A1 body and A1 enamel (0.5 mm each); total: 1.5 mm		
D1.0+E0.5	A1 dentin (1.0 mm) and A1 enamel (0.5 mm); total: 1.5 mm		
D1.0+B0.5	A1 dentin (1.0 mm) and A1 body (0.5 mm); total: 1.5 mm		
WD0.5+B0.5+E0.5	White dentin, A1 body and A1 enamel (0.5 mm each); total: 1.5 mm		
WD0.5+D0.5+E0.5	White dentin, A1 dentin and A1 enamel (0.5 mm each); total: 1.5 mm		
FL0.2+B0.8+E0.5	Flowable opaque (0.2 mm), A1 body (0.8 mm), and A1 enamel (0.5 mm); total: 1.5 mm		
FL0.2+D0.8+E0.5	Flowable opaque (0.2 mm), A1 dentin (0.8 mm), and A1 enamel (0.5 mm); total: 1.5 mm		
D1.5+E0.5	A1 dentin (1.5 mm) and A1 enamel (0.5 mm); total: 2 mm		
WD0.5+D1.0+E0.5	White dentin (0.5 mm), A1 dentin (1.0 mm), and A1 enamel (0.5 mm); total: 2 mm		
FL0.2+D1.3+E0.5	Flowable opaque (0.2 mm), A1 dentin (1.3 mm), and A1 enamel (0.5 mm); total: 2 mm		

RC discs of enamel (E), body (B), dentin (D), white dentin (WD) (Filtek Z350XT; 3M ESPE, St Paul, USA), and flowable opaque (FL) (IPS Empress direct Opaque; Ivoclar Vivadent AG, Liechtenstein) were obtained (n=10) by applying the RC into templates (1 mm, 1.5 mm, and 3 mm) made of polyvinyl siloxane impression material (Express XT Putty; 3M ESPE, St Paul, USA) according to each desired thickness, and pressed by thin glass slices, according to the desired RC thickness of each group (Table 1). Each increment was light activated with 1,200 mW/cm^2^ (Radii-cal LED curing light; SDI, Victoria, Australia) for 20 seconds at a 10 mm distance. The discs (Ø=10 mm) were ground and polished with silica carbide papers (SiC) of #600, #1200, and #2000 until achieving precisely the final desired thickness, without the presence of bubbles or surface failures. All discs were inspected by an optical microscope (Stereo Discovery V20; Carl Zeiss, Oberkochen, Germany) and if any surface defect was detected, the disc was replaced.

RC discs of each substrate shade (A1-reference, A3, A4, B3, C2, and C4) (Filtek Z350XT Dentin; 3M ESPE, St Paul, USA) were also obtained by using the same aforementioned procedures (Ø=10 mm × 3 mm). The color difference (∆E_00_) was measured by comparing the color coordinates L*, a*, b* of the RC layers (Table 1) over each discolored substrate with that of the reference: A1-shaded substrate (dentin) + A1 Body 0.5 mm + A1 Enamel 0.5 mm.

The color coordinates L*, a*, and b* was measured through a spectrophotometer (SP60; X-Rite, Michigan, USA), being L* from 0 (black) to 100 (white), a* for green (−a*) to red (+a*) and b* for blue (−b*) to yellow (+b*). It was followed by the CIE D65 Standard Illuminant and the CIE 2-degree standard observer for coordinate calculation. The applied test parameters were: spectral range of λ=400-700 nm at intervals of 10 nm, aperture setting of 8 mm, and 2 seconds of measuring time. To form the multilayer combinations, the RC discs (enamel, body, dentin, and flowable opaque) were overlapped according to each group (Table 1), always using a coupling solution (glycerol C3H803; Vetec Química Fina Ltda, Duque de Caxias, Brazil) to minimize light scattering between the layers, and keeping always the same polished surface of the last layer turned to the top. After each test, the specimens were cleaned with 78% isopropyl alcohol.

Each set was measured three times over the discolored substrates and a mean value for L*, a*, and b* was obtained; the same was done for the reference group. The color coordinates L*, a*, and b* were used to calculate the ∆E_00_ through the CIEDE 2000 formula ^22^, as follows:

$${\Delta E}_{00}={\left[{\left(\frac{∆L'}{K_{L}S_{L}}\right)}^{2}+{\left(\frac{∆C'}{K_{C}S_{C}}\right)}^{2}+{\left(\frac{∆H'}{K_{H}S_{H}}\right)}^{2}+R_{T}\left(\frac{∆C'}{K_{C}S_{C}}\right)\left(\frac{∆H'}{K_{H}S_{H}}\right)\right]}^{\frac{1}{2}}$$

where ΔL′, ΔC′, and ΔH′ are differences in luminosity (L′), chroma (C′), and hue (H′), respectively, to a pair of measurements. R_T_ is a rotation function that accounts for the interaction between chroma and hue differences in the blue region. S_L_, S_C_, and S_H_ are weighting functions that adjust the total ∆E_00_ for variation in the location of the color difference pair in the L^*^, a^*^, and b^*^ coordinates, and the parametric factors k_L_, k_C_, and k_H_ are correction terms for deviation from reference experimental conditions ^23^. The parametric factors were set as 1 ^24^.

The clinical implications adopted for the color difference findings were perceptibility threshold (PT) (∆E_00_ ≤ 0.81, excellent color matching) and acceptability threshold (AT) (∆E_00_ ≤ 1.77, acceptable color matching) ^25^.

The color coordinates L^*^, a^*^, and b^*^ of each discolored substrate were also measured, to be compared with the reference substrate (A1) through the CIEDE2000 formula.

Statistical tests of normality (Shapiro-Wilk) and homoscedasticity (Levene) were performed. Since all data was normally distributed, One-way ANOVA and Tukey post hoc tests (α=.05) were performed to evaluate the influence of the RC layering strategy on the ∆E_00_ for each substrate in comparison to the reference, with the use of statistical software (IBM SPSS Statistics for MacIntosh, v21; IBM Corp, New York, USA).

## Results

**Figure 1:**
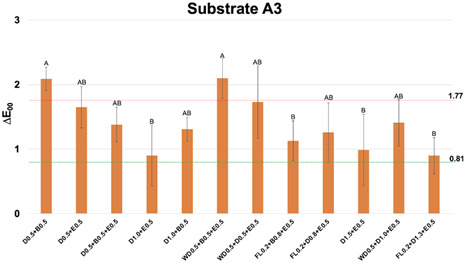
Mean and standard deviation values of CIEDE2000 color difference (∆E_00_) between each one of the multilayer resin composite strategy groups over substrate A3 and substrate A1, used as reference. Different letters show statistical differences (statistical test; p≤0,05). Perceptibility (0.81 ∆E_00_ units) and acceptability (1.77 ∆E_00_ units) thresholds were used for the analysis (33).

**Figure 2:**
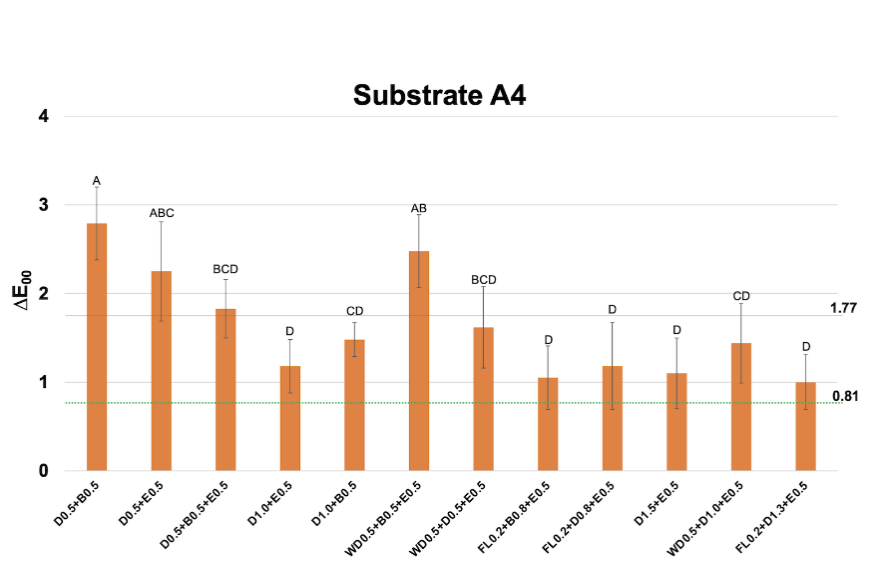
Mean and standard deviation values of CIEDE2000 color difference (∆E_00_) between each one of the multilayer resin composite strategy groups over substrate A4 and substrate A1, used as reference. Different letters show statistical differences (statistical test; p≤0,05). Perceptibility (0.81 ∆E_00_ units) and acceptability (1.77 ∆E_00_ units) thresholds were used for the analysis (33).

**Figure 3:**
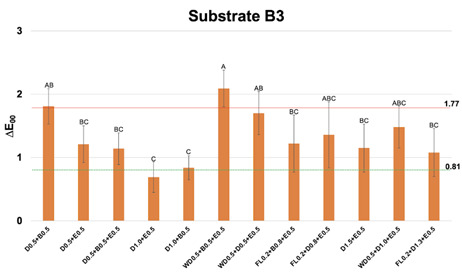
Mean and standard deviation values of CIEDE2000 color difference (∆E_00_) between each one of the multilayer resin composite strategy groups over substrate B3 and substrate A1, used as reference. Different letters show statistical differences (statistical test; p≤0,05). Perceptibility (0.81 ∆E_00_ units) and acceptability (1.77 ∆E_00_ units) thresholds were used for the analysis (33)

**Figure 4:**
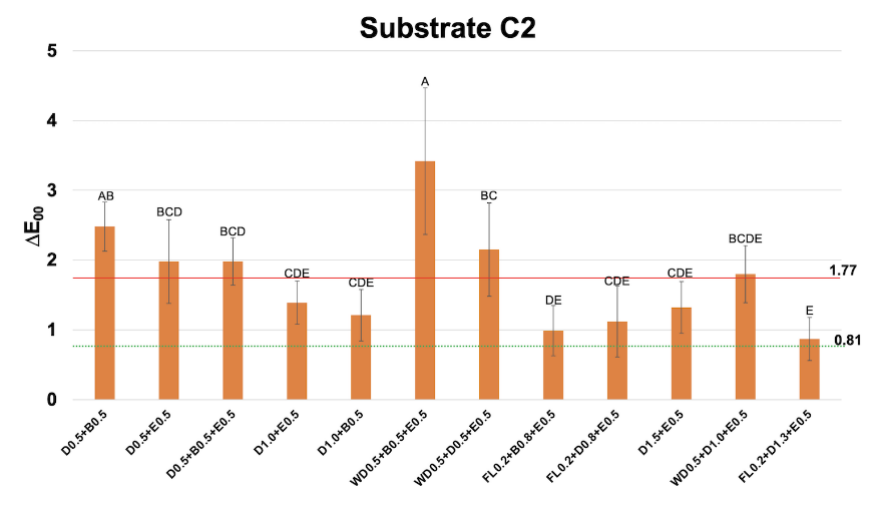
Mean and standard deviation values of CIEDE2000 color difference (∆E_00_) between each one of the multilayer resin composite strategy groups over substrate C2 and substrate A1, used as reference. Different letters show statistical differences (statistical test; p≤0,05). Perceptibility (0.81 ∆E_00_ units) and acceptability (1.77 ∆E_00_ units) thresholds were used for the analysis (33).

**Figure 5:**
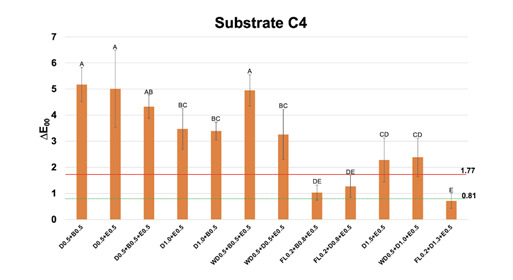
Mean and standard deviation values of CIEDE2000 color difference (∆E_00_) between each one of the multilayer resin composite strategy groups over substrate C4 and substrate A1, used as reference. Different letters show statistical differences (statistical test; p≤0,05). Perceptibility (0.81 ∆E_00_ units) and acceptability (1.77 ∆E_00_ units) thresholds were used for the analysis (33)

The RC layering strategy significantly influenced the ∆E_00_ over discolored substrates in comparison to the reference (*P*<0.001). ∆E_00_ values are depicted in graphical figures (Figure 1-5). All discolored substrates showed ∆E_00_ ≥ 1.77 in comparison to the reference A1 (Table 2), with C4 and A4 depicting higher values (∆E_00_ = 15.16 and 10.98, respectively).

**Table 2:** Mean values (standard deviation) of the L*, a*, and b* CIELab coordinates of the tested substrates. Color difference (∆E_00_) between discolored substrates in comparison with A1.

Substrate Shade	CIELab coordinates			∆E_00_
	L*	a*	b*	
A1	82.88 (2.1)	2.36 (0.1)	15.50 (0.9)	-
A3	76.16 (1.2)	6.98 (0.3)	24.27 (1.1)	7.51 (0.4)
A4	70.58 (1.6)	7.92 (0.3)	24.83 (1.0)	10.98 (0.6)
B3	74.72 (1.1)	6.64 (0.2)	27.21 (1.2)	8.75 (0.4)
C2	72.72 (1.3)	5.34 (0.3)	15.15 (0.9)	8.04 (0.5)
C4	62.70 (0.9)	3.26 (0.2)	16.75 (1.1)	15.16 (0.8)

In comparison to the reference, the outcomes for ∆E_00_ of the RC layering strategies over discolored substrates were as follows:

For the A3 substrate (Figure 1), almost all RC layering strategies showed ∆E_00_ below AT, including the 1 mm bilayer group D0.5+E0.5. The lower ∆E_00_ were obtained with groups of 1.5 mm (D1.0+E0.5 and FL0.2+B0.8+E0.5) and 2 mm (D1.5+E0.5 and FL0.2+D1.3+E0.5).

For substrate A4 (Figure 2), none of the RC layering strategies of 1 mm of thickness showed ∆E_00_ below AT. Most groups of 1.5 mm (except D0.5+B0.5+E0.5 and WD0.5+B0.5+E0.5) and all groups of 2 mm led to ∆E_00_ below AT.

Regarding substrate B3 (Figure 3), the group D1.0+E0.5 led to ∆E_00_ below PT. One group of 1 mm of thickness (D0.5+E0.5) provided ∆E_00_ below AT. The other groups of 1.5 mm (except WD0.5+B0.5+E0.5) and 2 mm led to ∆E_00_ below AT.

For substrate C2 (Figure 4), RC layering strategies of 1 mm of thickness did not show ∆E_00_ below AT. Other groups led to ∆E_00_ ≤ 1.77 for RC layering strategies of 1.5 mm of thickness (D1.0+E0.5, D1.0+B0.5, FL0.2+D0.8+E0.5, FL0.2+B0.8+E0.5) and 2 mm of thickness (D1.5+E0.5 and FL0.2+D1.3+E0.5).

For substrate C4 (Figure 5), ∆E_00_ values below PT were obtained with a trilayer RC layering strategy of 2 mm of thickness (FL0.2+D1.3+E0.5), and ∆E_00_ below AT was obtained for trilayer RC layering strategies of 1.5 mm of thickness (FL0.2+D0.8+E0.5 and FL0.2+B0.8+E0.5).

## Discussion

The layering strategy influenced the masking ability of RCs over discolored substrates as significant color differences were observed in comparison with the reference. Thus, the study hypothesis was accepted. These outcomes are attributed to differences in translucency and lightness among the RC layering strategies and by the variation in the final thickness of the combinations, which are considered major factors for masking discolored substrates ^2^ ^,^ ^9^ ^,^ ^10^ ^,^ ^11^ ^,^ ^12^ ^,^ ^20^.

Discolored substrates of varied shades were evaluated in the present study (A3, A4, B3, C2, and C4). According to the present findings, the most difficult substrate to mask was C4, as it was necessary the use flowable opaque RC as the first layer and, in consequence, the use of trilayers (RC combinations of 1.5 or 2 mm) (Figure 5). This is in accordance with previous studies that adopted the substrate C4 and reported that it is a challenging scenario to mask ^3^ ^,^ ^6^ ^,^ ^9^ ^,^ ^10^ ^,^ ^11^ ^,^ ^15^.

The RC restorations traditionally use dentin, body, and enamel layers. The combination of these materials is indicated to promote a natural aspect for direct restorations ^8^ ^,^ ^12^ ^,^ ^13^ ^,^ ^14^ ^,^ ^16^ ^,^ ^17^, through the presence of both opaque (dentin and/or body) and translucent (enamel) RCs. Such layers also mimic the lightness characteristics of the respective tooth structures. The use of such layers provided acceptable color matching for most discolored substrates evaluated (except C4). Increased thickness of the dentin layer was necessary, depending on substrate shade. The body RC is considered a universal material, since it presents intermediate translucency and lightness in comparison to enamel and dentin ^9^ ^,^ ^10^ ^,^ ^13^ ^,^ ^15^ ^)^ and, because of that, it might be used for several applications in the layering technique. However, in most of the evaluated combinations, the association of dentin and enamel RCs depicted lower color difference values than groups that contained the body as the substitute for one of them. This may be explained by the resulting optical characteristics of the combined RCs, whereas the dentin associated with the body generated an excessively opaque and brighter aspect, while the body associated with enamel generated a too translucent and darker result, promoting higher color differences in both situations ^13^ ^,^ ^14^ ^,^ ^21^.

The present study also evaluated white dentin and flowable opaque RCs as first layers. Color matching would be expected with the increase of the opacity and lightness, even in thin layers ^6^ ^,^ ^11^ ^,^ ^18^ ^,^ ^19^ ^,^ ^21^, which was observed with the use of flowable opaque RC for all discolored substrates tested. This might be attributed to the combination of high opacity and lightness of the first layer but also adequate thickness for the subsequent shaded RC layers placed over the flowable opaque RC ^6^ ^,^ ^9^ ^,^ ^11^. Moreover, opaque RCs with shades, such as the flowable RC adopted in this study, might have facilitated color matching in association with the subsequent layers ^21^. The use of white dentin RC as the first layer showed acceptable color matching for substrates A3, A4, and B3, when subsequent dentin and enamel layers were applied. It might be suggested that relevant light transmission occurs through the white dentin RC layer and, therefore, it would best serve as a chroma attenuating. In this sense, it would be clinically indicated for discolored substrates of a similar hue to that desired for the final restoration. It is also important to note that the subsequent use of body and enamel RC layers after white dentin did not provide ∆E_00_ below AT for any substrate, highlining the importance of a subsequent shaded dentin layer, which would help to attenuate the light reflectance from the substrate but also the whitish aspect of the white dentin layer ^18^ ^,^ ^21^. One possibility for clinical application would be the use of pigments over the white dentin to individualize its chroma, as desired for the case, before the application of the next layers.

Regarding the final thicknesses of the RC combinations, 1 mm, 1.5 mm, and 2 mm were evaluated. The group of 1 mm of thickness combining 0.5 of dentin + 0.5 mm of enamel provided acceptable color matching over substrates A3 and B3. Several RC layering techniques of 1.5 mm of thickness led to acceptable color matching, for all discolored substrates, presenting similar or even lower ∆E_00_ than some groups of 2 mm of thickness. The groups of 1.5 mm of thickness presented acceptable color matching even for substrate C4, using the flowable opaque RC as the first layer (Figure 5). This is in accordance with a previous scoping review that reported acceptable color matching over discolored substrates for RC groups of 1.5 mm of thickness containing at least one layer of opaque materials ^21^. The groups of 2.0 mm of thickness were effective in reducing color differences in comparison to those of 1 mm and 1.5 mm only for substrate C4, in which ∆E_00_ below PT was obtained with the use of flowable opaque RC; however, such increase in tooth preparation should be clinically evaluated to ensure that adequate structure is maintained.

Despite the findings of the present study, some limitations must be considered. The masking ability of the smoothly polished RC surface, adopted in the study, might be different from the characterized irregular surface of restorations, because of differences in light scattering. Additionally, only one final shade was tested; the findings might be different for other shades. The outcomes might also be different for RC materials of other companies. Even so, we believe that the present study was effective in showing that layering strategies are effective in masking discolored substrates when the proper thickness and layering strategies are used. Thus, when clinicians detect the discoloration level of the substrate, which can be made with experience and use of some equipment such as the VITA® shade guide for instance, it may be possible to define the best layering strategy to provide adequate masking for esthetic restorations.

## Conclusion

Within the limitations of this current study, it was concluded that:

The layering strategy influences the masking ability of resin composites over discolored substrates. In comparison to the reference: the 1 mm bilayer combining 0.5 mm of dentin and 0.5 mm of enamel produced acceptable color matching for substrates A3 and B3; the 1.5 mm bilayer applying 1.0 mm of dentin and 0.5 mm of enamel produced excellent color matching for substrate B3 and acceptable color matching for substrates A3, A4, and C2; for substrate C4, excellent color matching was obtained with a trilayer of 2 mm of thickness (0.2 mm of flowable opaque + 1.3 mm dentin + 0.5 mm enamel) and acceptable color matching with the trilayer of 1.5 mm of thickness (flowable opaque + 0.8 mm dentin or body + enamel).

## References

[1] John MT, Slade GD, Szentpetery A, Setz JM (2004). Oral health-related quality of life in patients treated with fixed, removable, and complete dentures 1 month and 6 to 12 months after treatment. Int J Prosthodont.

[2] Yanikian C, Yanikian F, Sundfeld D, Lins R, Martins L (2019). Direct composite resin veneers in nonvital teeth: A still viable alternative to mask dark substrates. Oper Dent.

[3] Spaveras A, Vjero O, Anagnostou M, Antoniadou M (2015). Masking the discolored enamel surface with opaquers before direct composite veneering. J Dent Oral Disord Ther.

[4] Bayne SC, Ferracane JL, Marshall GW, Marshall SJ, van Noort R (2019). The evolution of dental materials over the past century: Silver and gold to tooth color and beyond. J Dent Res.

[5] Ferracane JL (2011). Resin composite - state of the art. Dent Mater.

[6] Kim SJ, Son HH, Cho BH, Lee IB, Um CM (2009). Translucency and masking ability of various opaque-shade composite resins. J Dent.

[7] Dietschi D, Fahl N (2016). Shading concepts and layering techniques to master direct anterior composite restorations: An update. Br Dent J.

[8] Elgendy H, Maia R, Skiff F, Denehy G, Qian F (2019). Comparison of light propagation in dental tissues and nano-filled resin-based composite. Clin Oral Investig.

[9] Perez BG, Gaidarji B, Palm BG, Ruiz-López J, Pérez MM, Durand LB (2022). Masking ability of resin composites: Effect of the layering strategy and substrate color. J Esthet Restor Dent.

[10] Miotti LL, Santos IS, Nicoloso GF, Pozzobon RT, Susin AH, Durand LB (2017). The use of resin composite layering technique to mask discolored background: A CIELAB/CIEDE2000 analysis. Oper Dent.

[11] Lehr RM, Perez BG, Gaidarji B, Dalmolin A, Durand LB (2022). Masking ability of the combined application of opaquers and resin composite on discolored backgrounds. Oper Dent.

[12] Ismail EH, Dawson DV, Vidal CMP, Skiff FN, Brogden K, Maia RR (2023). The effect of varying dentin chroma and enamel thickness on the color of A2 double layered resin composite. Oper Dent.

[13] Kamishima N, Ikeda T, Sano H (2005). Color and translucency of resin composites for layering techniques. Dent Mater.

[14] Pecho OE, Ghinea R, Amaral EA, Cardona JC, Bona AD, Pérez MM (2016). Relevant optical properties for direct restorative materials. Dent Mater.

[15] Dalmolin A, Perez BG, Gaidarji B, Ruiz-López J, Lehr RM, Pérez MM (2021). Masking ability of bleach-shade resin composites using the multilayering technique. J Esthet Restor Dent.

[16] Betrisey E, Krejci I, Di Bella E, Ardu S (2016). The influence of stratification on color and appearance of resin composites. Odontology.

[17] Marjanovic J, Veljovic DN, Stasic JN, Savic-Stankovic T, Trifkovic B, Miletic V (2018). Optical properties of composite restorations influenced by dissimilar dentin restoratives. Dent Mater.

[18] Soares PM, Pereira GKR, Bacchi A (2023). Resin composite layering on discolored substrates ensures masking ability for monolithic ceramics. J Esthet Restor Dent.

[19] Dotto L, Machado PS, Slongo S, Pereira GKR, Bacchi A (2021). Layering of discolored substrates with high-value opaque composites for CAD-CAM monolithic ceramics. J Prosthet Dent.

[20] An JS, Son HH, Qadeer S, Ju SW, Ahn JS (2013). The influence of a continuous increase in thickness of opaque-shade composite resin on masking ability and translucency. Acta Odontol Scand.

[21] Perez BG, Gaidarji B, Righes DZ, Pecho OE, Pereira GKR, Durand LB (2023). Masking ability of resin composites: A scoping review. J Esthet Restor Dent.

[22] Salas M, Lucena C, Herrera LJ, Yebra A, Bona AD, Pérez MM (2018). Translucency thresholds for dental materials. Dent Mater.

[23] Luo MR, Cui G, Rigg B (2001). The development of the CIE 2000 color-difference formula: CIEDE2000. J Opt Soc Am A Opt Image Sci Vis.

[24] Commission Internationale de l´Eclairage (2004). CIE technical report: Colorimetry.

[25] Paravina RD, Pérez MM, Guinea R (2019). Acceptability and perceptibility thresholds in Dentistry: a comprehensive review of clinical and research applications. J Esthet Restor Dent.

