# Errata

**DOI:** 10.1590/0103-6440202405500er

**Published:** 2024-10-25

For the article published as:

Soares PM, Chiapinotto GF, DDS AB, Pereira GKR. Masking discolored substrates with resin composites: effect of layering strategies. Braz Dent J [Internet]. 2024;35:e24-5910. Available from: https://doi.org/10.1590/0103-6440202405910

Correcting the author's name

Atais Bacchi DDS

To 

Atais Bacchi

